# The Relationship Between Deviation Control and Accommodative Load in Patients with Intermittent Exotropia

**DOI:** 10.18502/jovr.v21.17126

**Published:** 2026-03-02

**Authors:** Pegah Behjati, Payam Nabovati, Mohammad Etezad Razavi, Abdollah Farzaneh

**Affiliations:** ^1^Rehabilitation Research Center, Department of Optometry, School of Rehabilitation Sciences, Iran University of Medical Sciences, Tehran, Iran; ^2^Eye Research Center, Mashhad University of Medical Sciences, Mashhad, Iran

**Keywords:** Accommodative Load, Deviation Control, Intermittent Exotropia

## Abstract

**Purpose:**

To investigate the relationship between deviation control and accommodative load in patients with intermittent exotropia (IXT).

**Methods:**

A total of 35 patients with IXT aged 5-21 years (average, 10.46 ± 4.42 years) with a best-corrected visual acuity of 20/20 or better were included in this study. All examinations, including binocular and monocular refraction, deviation angle, near stereoacuity, and deviation control, were done in patients with the best optical correction.

**Results:**

Of the 35 patients, 16 (45.7%) were male and 19 (54.3%) were female. The results indicated a statistically significant difference in the average distance and near deviation angles between good, relatively good, and poor deviation control levels (*P* = 0.012). The results also showed that the likelihood of deterioration in deviation control increased significantly with deviation angle (*P* = 0.012). The accommodative load of the right and left eyes at distance and near fixation did not differ significantly across the three levels of deviation control. Based on the results of univariate logistic regression, no statistically significant relationship was observed between accommodative load (both the right and left eyes at distance and near) and deviation control level. However, there was a significant difference between the accommodative loads of the left and right eyes at near and distance (*P* < 0.001).

**Conclusion:**

In patients with IXT, deviation control decreased with deviation angle, and accommodative load increased in near fixation compared to distance fixation, but differences were not significant across the three deviation control groups (good, relatively good, and poor).

##  INTRODUCTION

Strabismus is a condition characterized by misalignment of the eyes, leading to a deviation from a normal, parallel gaze.^[1]^ Exotropia is a form of strabismus that affects approximately 1% of the global population and is more common in the Middle East, South Africa, and Asia than in other regions.^[2,3,4,5]^ About 48% to 92% of patients with exotropia have intermittent exotropia (IXT).^[2,6,7]^ In IXT, one eye fixates on a target while the other points outward, especially when looking far away, when in bright sunlight, or when the person is tired or daydreaming.^[8]^

In the early stage of IXT, patients can maintain normal eye position and binocular visual function through the fusion and accommodative mechanisms. With disease progression, the fusion and accommodative convergence (AC) functions gradually weaken; as a result, exotropia becomes more frequent and occasionally constant.^[9]^

According to Burian's classification, patients with IXT can be divided into four groups: basic, divergence excess, pseudo-divergence excess, and convergence insufficiency.[8,10-12] The severity of IXT depends on three factors: the deviation angle, exodeviation control ability, and stereoacuity.^[1]^

Common treatments for IXT include spectacle correction of refractive error, patching, overminus lens therapy, orthoptic therapy, botulinum toxin injection, and surgical procedures.^[13]^

A major concern regarding surgery in children with IXT is overcorrection and postoperative esotropia, which are among the most important side effects of these procedures and could cause loss of fusional control and decreased stereoacuity.^[14]^ Therefore, proponents of nonsurgical methods insist on postponing surgery for as long as possible while engaging children in preoperative measurements to reduce the risk of such complications.^[15]^ Surgery is needed in the following cases: increased frequency of manifest deviations (more than once per day), a deviation angle of 15 prism diopters at distance (6 m), and complaints such as diplopia.^[16]^

In the presence of IXT, binocular fusion requires greater effort from the vergence system to produce convergence and compensate for exotropia so that the eyes are aligned.^[17,18]^ Since accommodation and convergence control are linked processes, this additional convergence likely induces an accommodative response. The accommodative convergence/accommodation (AC/A) ratio plays a key role in IXT classification and the adoption of an appropriate treatment method; hence, the significance of its clinical measurement needs to be considered.

Previous studies have mostly used dynamic retinoscopy to measure accommodative responses; however, this is a subjective method that depends on the examiner's ability and experience,^[19]^ and it is also incapable of measuring accommodative load.

Accommodative load is defined as the difference in refractive error between binocular and monocular viewing, assessed separately at distance and near fixation.^[18]^ This mechanism helps the vergence system control and maintain binocular vision, and it depends on the deviation angle, the amount of positive fusional vergence, the AC/A ratio, and the level of deviation control. However, the limited information available about this mechanism is largely derived from subjective measurements. By measuring accommodative load through objective methods, one can examine its role in controlling exotropia in patients with IXT more accurately.

In this study, Natural Vision Auto Ref/Keratometer NVISION-K5001 (Rexxam, Tokyo, Japan) was used to objectively measure accommodative load. This autorefractometer allows for open-field binocular fixation, which simulates natural human vision. This device allows measurement of refractive errors under different accommodative stimuli, in addition to calculating accommodation, convergence, and pupillary constriction data.^[16]^ Mohney and Holmes' Intermittent Exotropia Control scoring was used to assess the level of IXT control for both near and distance fixation.^[20,21]^

##  METHODS

The present study featured a cross-sectional, descriptive-analytical design. The study protocol was approved by the Ethics Committee of Iran University of Medical Sciences (ethics code: IR.IUMS.REC.1402.372). After obtaining ethical approval and the necessary permits to conduct the study, patients with IXT aged 5-21 years who met the inclusion criteria were selected. All patients underwent a complete ophthalmic examination by a fellow in strabismus. Cycloplegic refraction with cyclopentolate was used for patients who needed optical correction. The patients had the best optical correction during all examinations, including the measurement of binocular and monocular refraction, deviation angle, distance vision, and deviation control.

### Participants

The study population comprised the patients with IXT who were admitted to Dr. Etezad Razavi's Ophthalmology Clinic. We evaluated patients with basic exotropia who had a normal AC/A ratio. Patients with the best-corrected visual acuity of 20/20 or better were included in the study, while patients with amblyopia, anisometropia > 2 diopters, refractive errors above - 6.00 and +3.00 diopters, astigmatism > 1.50 diopters, isolated vertical deviation, vertical deviation > 5 prism diopters, A- and V-pattern, deep sensory adaptations (suppression, abnormal retinal correspondence), constant exotropia with no fusional response, ocular motility disorders, other diseases affecting vision, and history of eye surgeries were excluded.

#### Accommodative load assessment

The NVISION-K5001 open-field autorefractometer was used to measure refractive error under binocular and monocular conditions while the patient wore corrective glasses and looked at specific targets first at distance (6 m) and then at near (33 cm). To evaluate the accommodative load, refraction was performed in monocular and binocular conditions for each eye. The difference in refraction between binocular and monocular conditions was considered the accommodative load. The accommodative load was evaluated for each eye.

#### Sensory testing 

The hole-in-a-card test was performed to determine ocular dominance. In patients with IXT, normal distance stereoacuity is associated with good control of deviation, whereas reduced stereoacuity is associated with poorer control.^[22]^ Furthermore, early detection of abnormal stereoacuity (both near and, if possible, distance) and near fusional vergence amplitude may aid in determining the appropriate timing of surgery in patients with IXT.^[23,24]^ However, in this study, there was no access to the Frisby test or similar tests for evaluating distance stereoacuity. Therefore, only near stereoacuity was assessed using the TNO test.

To test sensory adaptations such as suppression and abnormal retinal correspondence, we employed the Worth 4 dot test with red/green glasses (when the patient needed correction) at distance (6 m) and at near (33 cm). Patients with constant suppression or with abnormal retinal correspondence were excluded from the study.

#### Deviation control measurement

Following the studies by Mohney and Holmes, we evaluated the level of deviation control using the scale described below:^[21]^

5 = Constant exotropia

4 = Exotropia > 50% of the exam before dissociation

3 = Exotropia < 50% of the exam before dissociation

2 = No exotropia unless dissociated, recovers in > 5 seconds

1 = No exotropia unless dissociated, recovers in 1-5 seconds

0 = No exotropia unless dissociated, recovers in < 1 second (phoria)

This scale is applied to each patient for both distance and near fixation. When combined, it yields an overall control score ranging from 0 to 10. Levels 5 to 3 are assessed during an initial 30-second period of observation. Levels 2 to 0 are graded as the worst of three rapidly successive trials. An occluder is placed over the right eye for 10 seconds and then removed to measure the time it takes for fusion to be re-established. The left eye is then occluded for 10 seconds, and the time to refusion is similarly calculated. A third trial of 10-second occlusion is performed, covering the eye that required the longest time to re-fuse. The worst level of control observed following the three 10-second periods of occlusion should be recorded for that visit.

Due to the small sample size and unequal distribution of deviation control across groups, we categorized participants into three groups based on their deviation-control status. According to the Mohney and Holmes criteria, a score of 0 to 2 was considered good control, 3 relatively good control, and 4 poor control.

**Table 1:** Descriptive statistics of the variables

**Variable**	**Mean ± SD**	**Range (Min to Max)**
Right eye spherical equivalent (D)	-0.80 ± 1.23	-4.75 to 0.50
Left eye spherical equivalent (D)	-0.73 ± 1.27	-5.38 to 0.25
Deviation angle at distance (PD)	23.94 ± 9.7	8 to 50
Deviation angle at near (PD)	24.94 ± 9.8	10 to 50
Stereopsis (S)	158.57 ± 97.5	30 to 480
Right eye accommodative load at distance (D)	-0.39 ± 0.55	0.00 to 3.00
Right eye accommodative load at near (D)	-0.42 ± 0.56	0.00 to 3.50
Left eye accommodative load at distance (D)	-0.92 ± 0.46	0.00 to 2.50
Left eye accommodative load at near (D)	-0.95 ± 0.51	0.00 to 2.25
D, diopter; PD, prism diopter; S, second.		

**Table 2:** ANOVA results for deviation angle and deviation control

**Variable**	**Deviation control level**	**Mean ± SD**	***P*** **-value**
Deviation angle at distance (PD)	Good	15.00 ± 4.76	
	Relatively good	20.54 ± 6.01	< 0.001
	Poor	30.33 ± 9.80	
Deviation angle at near (PD)	Good	15.25 ± 2.27	
	Relatively good	19.08 ± 5.58	0.012
	Poor	–	
PD, prism diopter.			

**Table 3:** Results of univariate logistic regression for deviation angle and deviation control

**Variable**	**Odds Ratio**	**95% CI**	**SE**	***P*** **-value**	**R^2^**
Deviation angle at distance (PD)	1.221	1.064 to 1.402	0.07	0.004	0.343
Deviation angle at near (PD)	1.114	1.230 to 1.009	0.05	0.032	0.159
PD, prism diopter.					

**Table 4:** ANOVA results for accommodative load and deviation control

**Variable**	**Deviation control level**	**Mean ± SD**	***P*** **-value**
Right-eye accommodative load at distance (D)	Good	0.13 ± 0.06	
	Relatively good	0.21 ± 0.14	0.207
	Poor	0.93 ± 0.47	
Left-eye accommodative load at distance (D)	Good	0.13 ± 0.06	
	Relatively good	0.19 ± 0.27	0.432
	Poor	0.97 ± 0.51	
Right-eye accommodative load at near (D)	Good	0.47 ± 0.39	
	Relatively good	0.65 ± 0.47	0.700
	Poor	-	
Left-eye accommodative load at near (D)	Good	0.43 ± 0.36	
	Relatively good	0.65 ± 0.47	0.584
	Poor	-	
D, diopter.			

**Table 5:** Results of univariate logistic regression for accommodative load and deviation control

**Variable**	**Odds ratio**	**95% CI**	**SE**	***P*** **-value**	**R^2^**
Right-eye accommodative load at distance (D)	2.611	0.423 to 16.097	0.93	0.004	0.343
Left-eye accommodative load at distance (D)	2.337	0.456 to 11.977	0.83	0.309	0.05
Right-eye accommodative load at near (D)	1.339	0.315 to 5.694	0.74	0.693	0.004
Left-eye accommodative load at near (D)	1.532	0.341 to 6.884	0.77	0.578	0.008
D, diopter.					

**Table 6:** Results of paired *t*-test for near and distance accommodative loads for each eye

**Variable**	**Mean**	**SD**	***P*** **-value**
Right-eye accommodative load at distance (D)	-0.39	0.55	< 0.001
Left-eye accommodative load at distance (D)	-0.92	0.46	
Right-eye accommodative load at near (D)	-0.42	0.56	< 0.001
Left-eye accommodative load at near (D)	-0.95	0.51	
D, diopter.			

#### Deviation angle measurement

The deviation angle was measured using the alternate prism cover test (APCT) at 6 m and 33 cm.

### Statistical Analysis

Statistical analysis was performed in SPSS version 23 (IBM Corp, Armonk, NY, USA). First, the normality of the data was assessed using the Shapiro-Wilk test and normality curves. We also recorded descriptive statistics, including mean and standard deviation, for the variables. Based on the level of deviation control, patients were divided into three groups: good control (0 to 2 on the Mohney-Holmes scale), relatively good control (3 on the Mohney-Holmes scale), and poor control (4 on the Mohney-Holmes scale). The average responses and accommodative loads were compared across the groups using an independent-samples *t*-test. Simple and multivariate logistic regression models were used to investigate the relationship between response, accommodative load, and deviation control while controlling for confounding variables. Finally, odds ratio (OR) values and 95% confidence intervals were presented. *P* < 0.05 was considered statistically significant.

##  RESULTS

After the inclusion/exclusion criteria were applied, 35 individuals with an average age of 10.46 ± 4.42 years (range, 5 to 21 years) were included in this study. Of these, 16 (45.7%) were male, and 19 (54.3%) were female. The spherical equivalent data for the right and left eye, deviation angle at near and at distance, stereopsis, right eye accommodative load at near and at distance, and left eye accommodative load at near and at distance are reported in Table 1.

In terms of deviation control at distance, among 35 patients, 4 (11.4%) had good control, 13 (37.1%) had relatively good control, and 18 (51.4%) had poor control. In terms of deviation control at near, 27 patients (77.1%) had good control, 8 (22.9%) had relatively good control, and none had poor control. Regarding the dominant eye, 23 patients (65.7%) were right-eye dominant and 12 (34.3%) were left-eye dominant.

Based on the results of the analysis of variance (ANOVA) shown in Table 2, the average deviation angles at distance and near for the three levels of deviation control (good, relatively good, and poor) indicate a statistically significant difference.

Univariate logistic regression was used to investigate the effect of deviation angle on deviation control. The results show that the model is statistically significant. In other words, the likelihood of deterioration in deviation control increases significantly with increasing deviation angle. The results of logistic regression and OR values are reported in Table 3.

ANOVA was used to compare the average accommodative load between different levels of deviation control. As shown in Table 4, there is no statistically significant difference between the three levels of deviation control in the accommodative load of the right and left eyes at distance and near. Univariate logistic regression was also used to investigate the relationship between accommodative load and deviation control. Based on the results shown in Table 5, no statistically significant relationship was observed between these variables.

Near and distance accommodative loads were also compared for each eye using a paired *t*-test, and the results indicate a significant difference [Table 6].

##  DISCUSSION

There have been studies on accommodative load in patients with IXT and its effects on deviation control; however, these studies have limitations, including insufficient attention to deviation-control classification systems and a narrow age range. Therefore, due to the importance of the deviation control process in patients with IXT, its contributing factors should be investigated more thoroughly. Moreover, it is important to conduct further studies in patients with IXT due to the many uncertainties that exist regarding the suitable time for surgery, deviation control methods, the role of accommodation, and the effect of stereopsis and deviation angle on deviation control.

In this study, the role of accommodative load in deviation control among patients with IXT was investigated using the NVISION-K5001 open-field autorefractometer. This device enables measurement of refractive errors under open-field binocular and monocular conditions that simulate natural human vision. Using this device, the monocular and binocular refractions of patients with IXT at near and distance were calculated with optical refraction correction, and the amount of accommodative load was measured according to the model presented in the methodology section.

The results showed that the average deviation angle at distance and near was significantly different between the three levels of good, relatively good, and poor deviation control. However, no significant differences in accommodative load were observed among the three levels of deviation control. There was also no significant difference between the three groups in terms of stereopsis in near fixation.

In this study, the likelihood of deterioration in deviation control increased with increasing deviation angle, both at distance and near fixation. Similarly, Gökgöz Özişik et al investigated the relationship between clinical factors and deviation control in patients with IXT and found that the greater the deviation angle, the weaker the deviation control.^[25]^ Another study by Superstein et al also supports the hypothesis that deviation control weakens as the deviation angle increases.^[26]^

In this study, stereopsis was evaluated at near fixation using the TNO test, and the results indicated no significant difference between the good and relatively good control groups (no patients had “poor control” at near fixation). Kang and Lee investigated the relationship between the degree of deviation control, near and distance stereoacuity, and surgical success in basic IXT. The patients were divided into three deviation control groups: good, fair, and poor. The results indicated significant differences in stereoacuity across the three groups. Between-group comparisons revealed a significant difference in stereoacuity between the good and poor control groups, and between the fair and poor control groups; however, the difference between the good and fair control groups was not statistically significant.^[27]^ Prior research has also shown that exotropia mainly affects stereoacuity at distance.^[22,24]^

In this study, accommodative load at near was higher than at distance, irrespective of deviation control level, while accommodative load did not differ significantly across deviation control groups. In the study by Ha et al on patients with basic IXT, the amount of accommodative load required to maintain binocular fusion was calculated at 6 m, 50 cm, 33 cm, and 20 cm. The results indicated that accommodative load in these patients is greater at near fixation compared to healthy controls.^[18]^

Ha et al^[18]^ used a control group and reported an increase in accommodative load at near fixation. In our study, the results showed that without separating groups based on deviation control, accommodative load is higher at near than at distance, which is consistent with the study by Ha et al. However, it must be noted that such an increase in accommodative load cannot be directly interpreted as an improvement in binocular performance. There are more accommodative efforts at near than at distance for two reasons: (1) the higher ability of the accommodation system and the relationship between convergence and accommodation; and (2) the existence of proximal accommodation. Therefore, an increase in accommodative load at near fixation in patients who try to control IXT deviation is a reasonable finding, but a significant relationship is expected between the increase in accommodative load and the level of deviation control, which was not observed in our study. In fact, there was no significant difference in right- and left-eye accommodative load (at distance and near) across good, relatively good, and poor control groups. An issue of note is that at near fixation, 27 patients had good control, 8 had relatively good control, and none had poor control. On the other hand, at distance fixation, 4, 13, and 18 patients exhibited good, relatively good, and poor control, respectively. This indicates that the increase in the accommodative load at near compared to distance can be attributed to better deviation control at near. However, apart from accommodation, two observations are worth noting: first, the ability of the vergence system is greater at near than at distance; second, as mentioned earlier, we did not find a significant difference across the three groups in terms of accommodative load.

In patients with IXT, increasing the angle of deviation is expected to increase the accommodative load, thereby improving control of the deviation. Based on the logistic regression analysis, deviation angle significantly affects deviation control, but there is no statistically significant relationship between deviation control and accommodative load. The deviation control score worsens with the increase in deviation angle. It seems that factors other than the angle of deviation can affect the accommodative load. In patients with a high angle of deviation, additional accommodation in binocular conditions alone could not improve deviation control. It seems that the accommodative load cannot be higher than a certain limit, which is why increasing the accommodative load is not enough to enhance convergence and, thereby, further improve deviation control.

In patients with IXT, usually one eye is the fixator or dominant eye, and the other is deviated or non-dominant. One of the aims of this study was to explore whether the accommodative load is different in the dominant eye compared to the nondominant eye. It would be more informative if we compared the accommodative load in fixating and deviating eyes. However, several participants in this study were young children, and some could not understand the directions during dominant-eye testing. Therefore, we could not determine ocular dominance in all participants and, as a result, separately evaluated the accommodative load in the right and left eyes.

An important issue is that most previous studies have included a control group to investigate and compare accommodative load between patients with IXT and controls, suggesting that accommodative load is higher in patients than in controls. However, the present study did not include a control group; instead, a sample of patients with IXT was divided into three groups based on deviation control: good, relatively good, and poor. As noted earlier, there was no significant difference in accommodative load across these groups.

In summary, in patients with IXT, deviation control decreases with deviation angle, and accommodative load increases at near fixation compared to distance. However, the difference between the three deviation control groups (good, relatively good, and poor) is not significant.

##  Financial Support and Sponsorship

This project was supported by the Iran University of Medical Sciences.

##  Conflicts of Interest

None.
